# When gold can do what iodine cannot do: A critical comparison

**DOI:** 10.3762/bjoc.7.97

**Published:** 2011-06-22

**Authors:** Sara Hummel, Stefan F Kirsch

**Affiliations:** 1 Department Chemie and Catalysis Research Center, Technische Universität München, Lichtenbergstr. 4, 85747 Garching, Germany

**Keywords:** catalysis, cycloisomerizations, domino reactions, gold, iodine

## Abstract

Gold catalysis has emerged as one of the most dynamic fields in organic synthesis. Only recently, more and more domino processes, for which gold pre-catalysts were found to be outstandingly effective, were paralleled by employing iodine electrophiles in place of gold compounds. This review highlights how, in certain cases, iodonium activation can match gold-catalyzed reactions to construct identical product scaffolds. Likewise, processes are discussed where mostly identical starting materials are transformed into diverse frameworks depending on whether gold or iodonium activation was used to trigger the reaction.

## Introduction

Over the past decade, the diverse reactivity of carbophilic Lewis acids has attracted considerable interest in the development of domino reactions [1–5] that are initiated by the catalytic activation of π-systems [6–13]. In particular, the utilization of gold pre-catalysts has led to numerous elegant contributions in both heterocycle and carbocycle syntheses [14–20]. In general, these processes are easy to perform under simple reaction conditions: Significant redox chemistry is not involved. Since gold complexes show outstanding functional group tolerance, there has also been a considerable increase in the applications of such complexes in target-oriented synthesis [21–26].

**Scheme 1:**
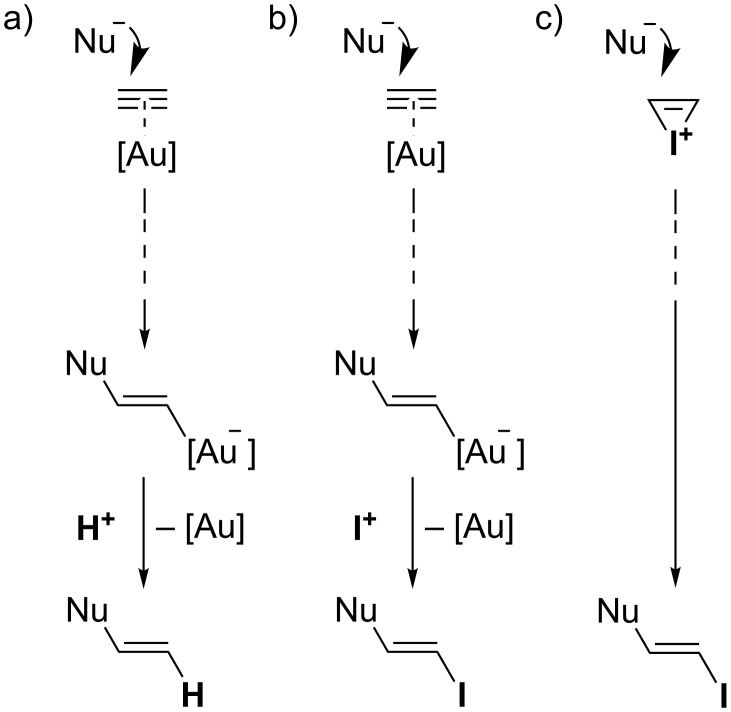
Mechanistic scenarios for alkyne activation.

A simplified mechanistic scenario for domino processes initiated by gold-catalyzed alkyne activation is depicted in Scheme 1a. After nucleophilic attack at the gold-activated alkyne and subsequent reorganization steps, the final step typically is a protodeauration [27–30] of the vinylgold intermediate to regenerate the catalytic species. In an analogous way, vinylgold intermediates can be successfully trapped by iodine electrophiles (and other electrophiles) to incorporate I rather than H in the final product (Scheme 1b) [27,31–40]. Even though both processes catalyzed by gold give rise to the same scaffolds, iodine incorporation allows for a further functionalization of the scaffold by classical cross-coupling reactions [41–42]. As a logical extension, one might speculate about analogous processes triggered by direct iodonium activation in the absence of gold catalysts (Scheme 1c). Since Barluenga, Larock, and others have shown over the last decades that various cyclizations of carbon and heteroatom nucleophiles with tethered alkynes can be accomplished by using simple iodine electrophiles [43–54], it would be of great interest to know to what extent transition metal-free processes can be substituted for gold-catalyzed processes.

This review is intended to demonstrate that, in some cases, gold-catalyzed domino processes can be paralleled by employing iodine electrophiles. In particular, if classical cationic intermediates are assumed to explain the gold-catalyzed reactivity of a substrate, it is reasonable to expect analogous reactivity for this substrate in the presence of electrophilic iodine. The reader will also realize how gold-catalyzed processes, which mechanistically benefit from the carbenoid character [55–58] of the reactive intermediates, cannot be matched by electrophilic processes. As highlighted in the discussion, a starting substrate can be transformed into diverse product classes, depending on whether gold or iodonium activation was used to trigger the transformation.

Since a comprehensive discussion on gold catalysis is not intended, the following examples of gold-catalyzed reactions simply illustrate certain prototype reactivity that i) is matched by electrophilic activation modes, or ii) leads to different product frameworks on treatment with electrophiles. The focus is put on alkyne activation only, while related processes based on the activation of alkenes and allenes are not covered in this review.

## Review

### Heterocyclization

The vast majority of gold-catalyzed cyclization modes have involved initial carbon-heteroatom bond formation [7,10]. As a general rule, the nucleophilic attack of a carbonyl (or imine) group onto an alkyne activated by gold-complexation generates first an oxonium (iminium) ion species, the cationic character of which then defines its follow-up chemistry [59–62]. Consequently, these reactions also have the potential to proceed in an analogous manner with classical iodine-based electrophiles. The same is true for intramolecular additions of simple heteroatom nucleophiles with protons attached. Nevertheless, heteroatom nucleophiles having no protons attached react in gold-catalyzed carboalkoxylations [63–66] and related processes where the analogous electrophilic processes are unknown. Catalyzed propargylic ester rearrangements [67–68] also remain the realm of gold-complexes since such reactions have not, so far, been achieved with classical electrophiles.

#### Analogous product formation

**Scheme 2:**
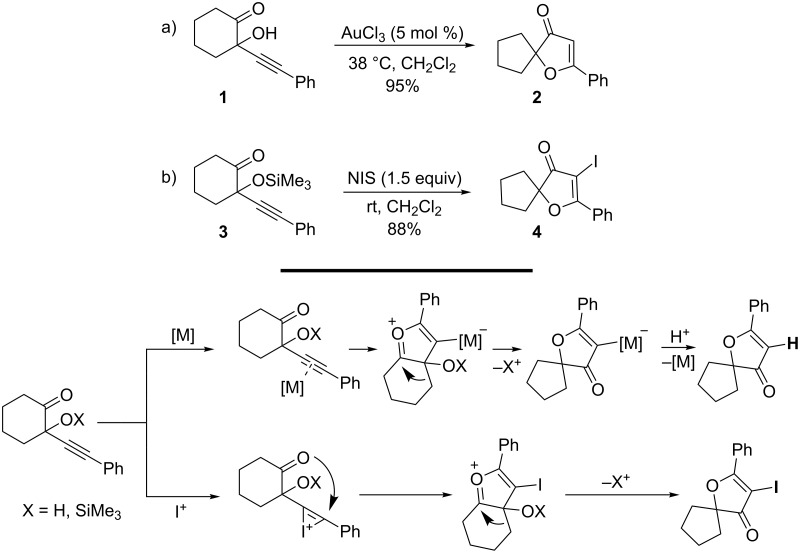
Synthesis of 3(2*H*)-furanones.

The synthesis of 3-furanones from 2-hydroxy-2-alkynyl carbonyl compounds is a striking example of how simple a gold-catalyzed pathway can be replaced with an iodonium-mediated one with both methods yielding the same core unit. In 2006, a substantial variation to the traditional synthesis of furanones was developed by Kirsch and co-workers from readily accessible 2-hydroxy-2-alkynylcarbonyl compounds by a gold-catalyzed cycloisomerization approach [69]. The gold-catalyzed cyclization of **1,** containing a hydroxy function at the propargylic position, was shown to undergo a cascade involving heterocyclization after activation of the alkyne π-system by the catalysts and a 1,2-alkyl shift (Scheme 2). It was found that this reaction was limited to aryl substituents on the alkyne when the reaction was catalyzed with AuCl_3_ at 38 °C in CH_2_Cl_2_. With alkyl substituents on the alkyne, the same reaction led to low yields of products and decomposition. Significantly, the reaction also proceeds with cyclic carbonyl compounds with seven- and eight-membered rings to give six- and seven-membered spirocycles, respectively. Consequently, Kirsch and co-workers described a reaction process using *N*-iodosuccinimide (NIS) instead of AuCl_3_ in an attempt to obtain 4-iodofuranones via an analogous iodonium-mediated cyclization [70]. With NIS as the electrophile at room temperature in CH_2_Cl_2_, the iodofuranone **4** was obtained in 88% yield. In contrast to the AuCl_3_ catalyzed cyclization, the iodonium-induced cascade tolerates the presence of aryl, alkenyl and alkyl groups on the alkyne. Of particular significance, however, is the fact that the reaction with NIS did not proceed with acyclic substrates, since in these cases no product formation occurred. In contrast, AuCl_3_ induced cyclization furnished acyclic products, albeit in low yields. Unlike gold-catalyzed heterocyclization, the NIS-mediated reaction allows access to C4-substituted products and therefore to fully substituted 3(2*H*)-furanones. It was deduced that a cyclic oxonium ion is produced in the first stage of the cascade via both gold- and iodonium-triggered cyclization. In both cases, the heterocyclization is followed by a 1,2-migration onto the oxonium ion, where the only difference is whether the final product bears a H or an I atom at the C4-position. It should be further noted that, in the case of the gold-catalyzed process, an external proton source (such as water) is required in the case of substrates with a silyl-protected tertiary hydroxyl group. Otherwise protodemetallation cannot occur to regenerate the catalytically active gold species. In the NIS-mediated pathway towards iodofuranones, the presence of a proton source is of no importance since both silyloxy and hydroxy substituents are reactive under the conditions.

**Scheme 3:**
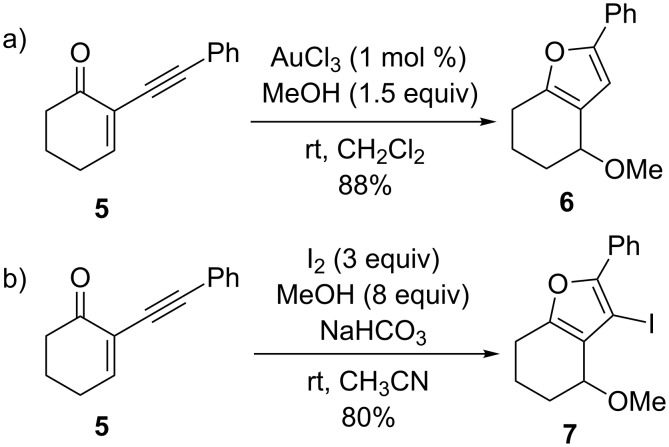
Synthesis of furans.

Another example where the iodine-induced reaction nicely parallels the gold-catalyzed reaction pathway was reported by Larock and co-workers in 2005 [71–72]. Thus, the reaction of 2-(1-alkynyl)-2-alken-1-one **5** catalyzed by AuCl_3_ affords the trisubstituted furan **6** in good yield (Scheme 3): A survey of other transition metal salts demonstrated that the optimum catalyst in CH_2_Cl_2_ at room temperature is AuCl_3_ based on reaction time and yield. Iodine, NIS, and PhSeCl were all shown to be useful electrophiles in the analogous transition-metal-free process.

**Scheme 4:**
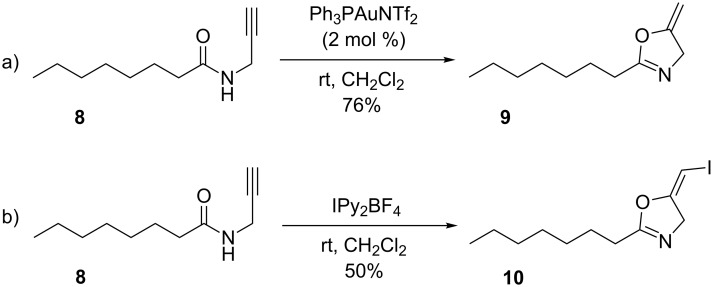
Formation of dihydrooxazoles.

Hashmi and co-workers demonstrated in 2010 that dihydrooxazole derivatives can be formed via both gold-catalyzed and iodonium-initiated pathways (Scheme 4) [34]. Interestingly, the bis(pyridine)iodonium tetrafluoroborate reagent [73] (IPy_2_BF_4_) developed by Barluenga proved to be the best reagent for the iodination pathway.

#### Diversity-creating transformations

**Scheme 5:**
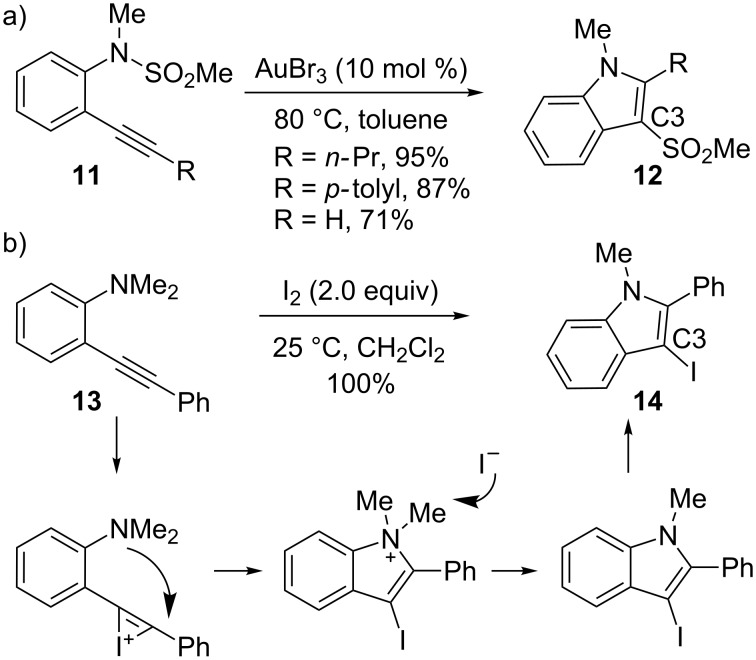
Variation on indole formation.

In contrast to the examples discussed above, the heterocyclization onto activated alkynes can generate quite different product structures when there are no protons present on the nucleophilic group. A variety of gold-catalyzed transformations that proceed through heterocyclization have been described over the last few years. For example, in 2007, Yamamoto and co-workers reported that when the heteroatom is substituted with a sulfonyl group, migration of the sulfonyl group occurs in an intramolecular fashion (Scheme 5a) [74]. The migration step is now well understood: The heteroatom can effectively stabilize the positive charge that develops. The coordination of gold to the triple bond of **11** and subsequent nucleophilic attack of the nitrogen atom leads to an onium ion intermediate, from which the sulfonyl group migrates intramolecularly to the metallated C3-position, to generate the sulfonylindole product **12**.

For this unique cyclization process, there has been no reported equivalent iodonium-mediated reaction. However, Larock and co-workers also investigated a cyclization process where the proton on the heteroatom is absent (e.g., **13→14**) [75]. After activation by the electrophile, the iodine substituent remains in the indole framework (Scheme 5b) since the 1,3-migration step observed in the gold-catalyzed reaction does not occur via electrophilic activation. Instead, due to the iodide ion still present in the reaction mixture, the ammonium ion intermediate undergoes S_N_1 or S_N_2 substitution, or even E2 elimination of an alkyl group. This system yields products in up to quantitative yields, and successfully displays the possibility of diverse product creation through the use of either gold- or iodonium-triggered heterocyclizations.

### Carbocyclizations with 1,5-enynes

Within the rapidly developing area of gold-catalysis, enyne cycloisomerizations have been particularly well studied [76–83]. With an appropriate substitution pattern, both 1,5- and 1,6-enynes can be transformed into a broad array of product scaffolds. The corresponding electrophilic transformations are far less developed. With the exception of an early report by Barluenga and co-workers [84], iodonium-induced carbocyclizations have been mainly restricted to the intramolecular arylation of alkynes (i.e., arene nucleophiles) [85–88] whilst simple olefins have been rarely used in this way.

#### Analogous cyclization modes

**Scheme 6:**
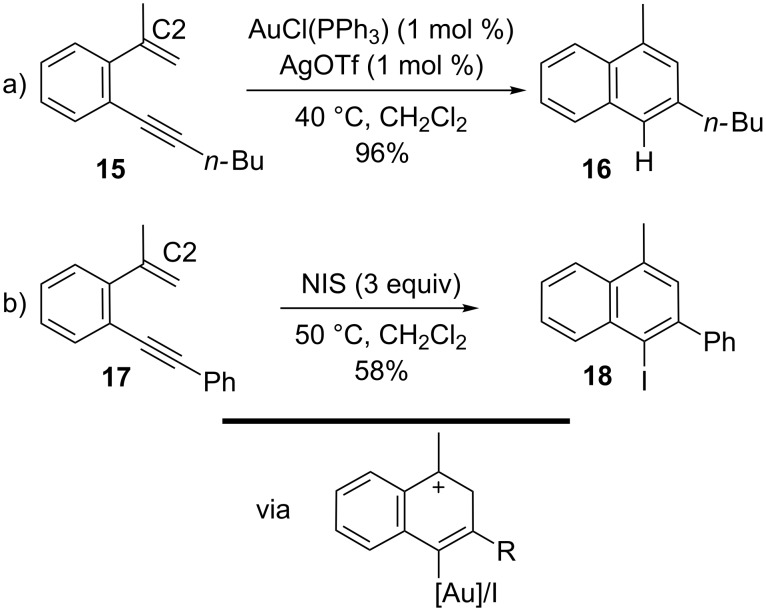
Formation of naphthalenes.

Several processes involving the cycloisomerization of 1,5-enynes have been realized in an analogous manner either by using gold-catalysts or electrophilic iodine sources. Typically, these processes are easily understood by postulating stabilized cationic intermediates. For example, aromatic 1,5-enynes can be cyclized to the corresponding naphthalenes in the presence of gold(I) catalysts as demonstrated by Shibata and co-workers in 2006 [89]. By using 1 mol % of both AuCl(PPh)_3_ and AgOTf at 40 °C in CH_2_Cl_2_, the 6-endo product was obtained exclusively, regardless of the nature of the counter ion of the Ag salt (Scheme 6). An analogous ring-closure was realized by Kirsch and co-workers in 2010, where NIS was used as the electrophilic agent [90]. Alkyne activation yields the iodonaphthalene as the sole product by an analogous 6-endo process. Both methods, regardless of whether the cyclization is triggered by Au^+^ or I^+^, require an alkyl substituent at the C2-position for naphthalene formation to proceed over indene formation, since it is required to stabilize the intermediate carbocation. As before, the only difference in the product structure is whether H or I has been incorporated.

**Scheme 7:**
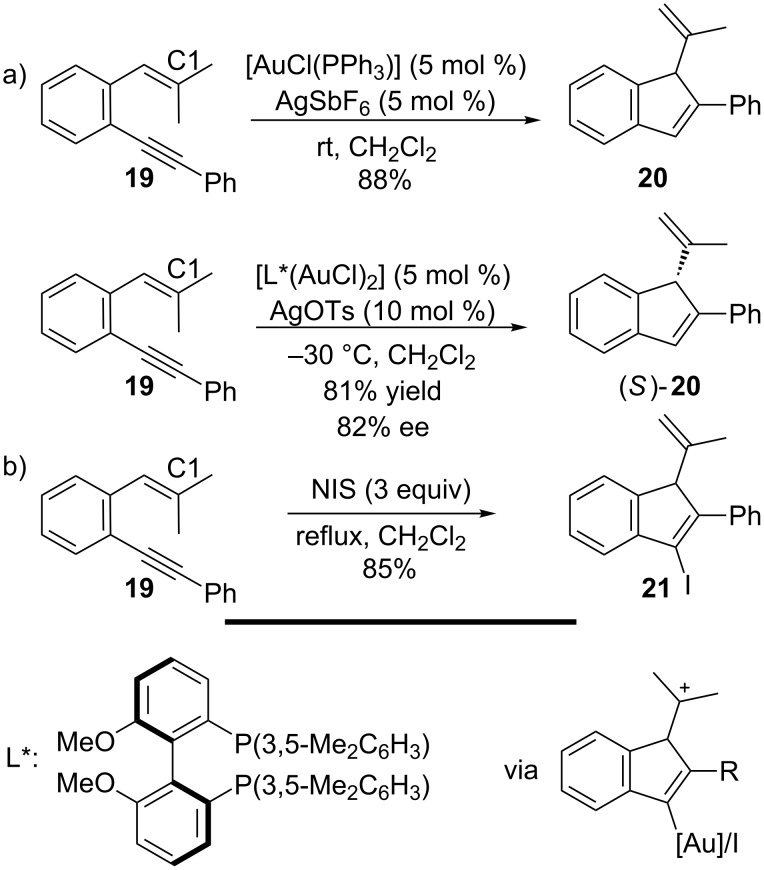
Formation of indenes.

Sanz and co-workers reported in 2010 that indenes were formed from *o*-alkynylstyrenes in the presence of AuCl(PPh_3_) activated by AgSbF_6_ (Scheme 7) [91]. A 1,5-enyne disubstituted at the terminal carbon of the alkene (e.g., **19**) leads to a very selective 5-endo carbocyclization where no side products resulting from 5-exo or 6-endo modes were observed. A mechanism was proposed involving a cationic intermediate after cyclization which, after loss of a proton and protodemetallation, afforded the product in 88% yield. The reaction can also be made to proceed enantioselectively by means of a gold complex with chiral ligands. The ligand *(S)-*3,5-xylyl-MeO-biphep (L*) gave the best results with respect to enantioselectivity, although all ligands tested with the gold complexes and silver salt AgSbF_6_ allowed full conversion to the indene. From these results, Sanz and co-workers also reported the corresponding halocyclization of *o*-alkynylstyrenes to yield 3-halo-1*H*-indenes by employing NIS as the iodonium source (Scheme 7b) [92]. This reaction proceeds via an exceptional 5-endo halocyclization, which likely results from the stability of the tertiary carbocation, since only H at the C1-position rendered the enyne unreactive under the conditions used (excess NIS, CH_2_Cl_2_ at reflux). From a synthetic point of view, the development of an asymmetric halocyclization towards indenes remains an ongoing task [93–96].

**Scheme 8:**
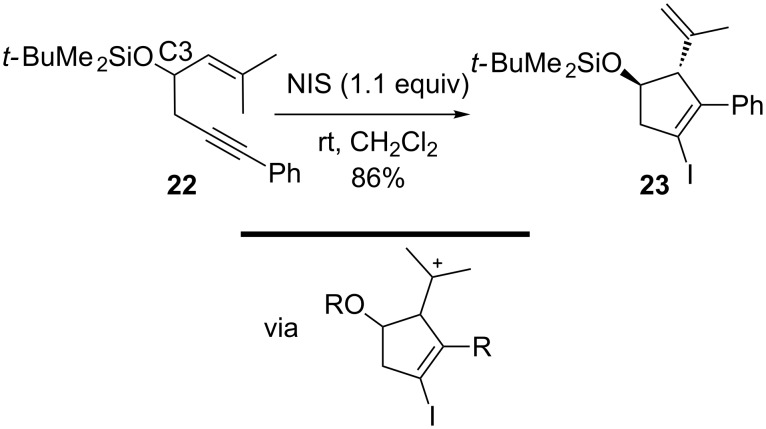
Iodocyclization of 3-silyloxy-1,5-enynes.

Notably, 1,5-enynes that do not contain an aryl system react in quite a similar manner when disubstituted at C1. For example, Michelet and co-workers reported the diastereoselective cycloisomerization of 1,5-enynes via a 5-endo mechanism triggered by iodine electrophiles (Scheme 8) [97]. With 1.1 equiv of NIS at room temperature in dichloromethane, full conversion of **22** occurred to yield selectively only the 5-endo product **23** in 86%. However, when there was no substituent such as the silyloxy group on C3, the reaction yield was considerably reduced. Again in this case, product formation is best understood by assuming the intermediate formation of the most stabilized carbocation. Surprisingly, the analogous gold-catalyzed process has not been described up to now; instead, 3-silyloxy-1,5-enynes with a stabilizing substituent at C2 were found to undergo a cascade consisting of 6-endo cyclization and a subsequent pinacol-type shift [37,98].

**Scheme 9:**
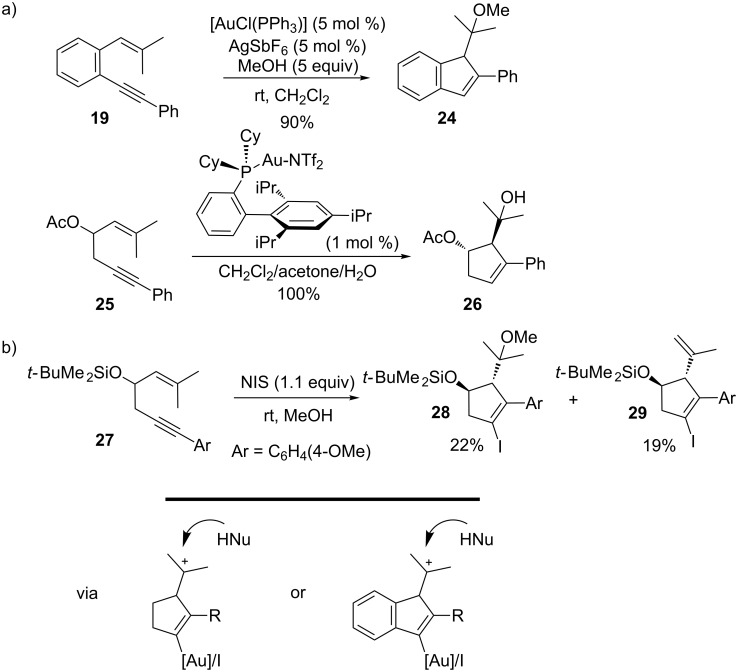
5-Endo cyclizations with concomitant nucleophilic trapping.

Since the 5-endo cyclizations discussed above most likely proceed through cationic intermediates, external nucleophiles were shown to trap these intermediates at C1 in both gold and iodonium-catalyzed reactions. Accordingly, Sanz and co-workers extended their investigations into 5-endo carbocyclizations of *o*-alkynylstyrenes by adding 5 equiv MeOH to their previous reaction conditions with Au-complexes and obtained the methoxy substituted product **24** at very high selectivity and 90% yield (Scheme 9a) [91]. Labeling experiments showed that the proposed mechanism where the cation is trapped by the nucleophile and subsequent loss of the proton and protodemetallation is viable. A more elaborate study of 5-endo hydroxy- and alkoxycyclizations of 1,5-enynes was described by Gagosz and co-workers as they examined a valuable route to functionalized cyclopentenes [99]. By the use of internal alkynes in substrates such as **25**, they were able to induce stereoselective cyclization followed by nuclophilic trapping. It was reported that by using water rather than methanol as the external nucleophile, excellent yields of the corresponding alcohol product **26** were obtained. Alcohols, for example methanol, and even acetic acid could also be employed as nucleophiles in the catalytic system. For this cascade, a gold carbene intermediate was postulated, although product formation can be explained well via a cationic intermediate as shown in Scheme 9. In an analogous way, Michelet and co-workers also showed that the NIS-mediated pathway can be combined with a nucleophilic trapping by using methanol (Scheme 9b) [97]. The reaction requires substituents at C1 and on the alkyne but, unlike in the case with gold-catalyzed cyclizations, the competing elimination through loss of a proton could not be entirely prevented. For example, a methoxyiodocarbocyclization of **27** occurred in 22% yield when the iodocyclization was run in methanol.

**Scheme 10:**
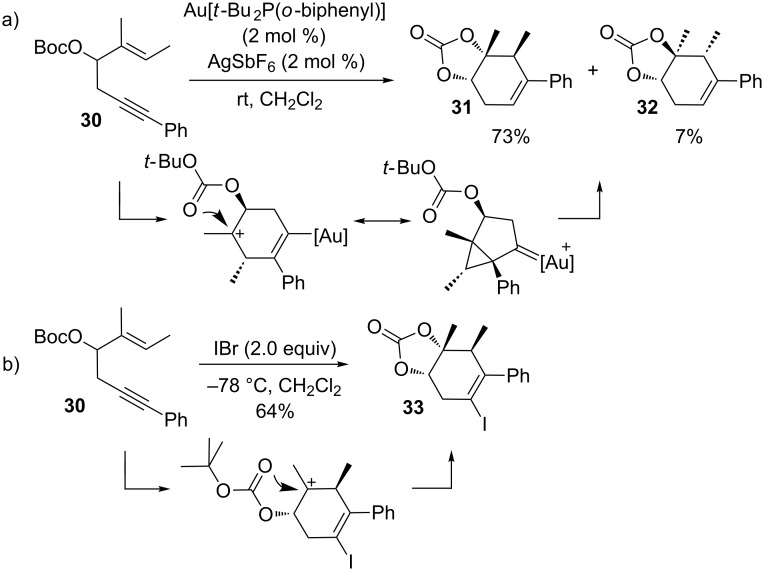
Reactivity of 3-BocO-1,5-enynes.

Additionally, the developing positive charge can be trapped with internal nucleophiles. For example, Shin and co-workers reported a valuable transformation using the *tert*-butoxycarbonyl (Boc) group to trap the cation following the activation of π-systems by gold in a route to cyclic carbonates (Scheme 10a) [100]. The Boc-group was considered to be very effective at undergoing intramolecular nucleophilic attack of the cationic intermediate to gain access to highly functionalized cyclohex-4-ene-1,2-diol derivatives from simple 3-BocO-1,5-enynes. A particular feature of this cyclization is that it developed good diastereoselective control over the adjacent stereocenters, including a quaternary carbon. As in the previous examples, the gold-catalyzed cyclization benefits strongly from a stabilizing substituent at C2 to direct the 6-endo mode of cyclization. Subsequent to the success of the gold-catalyzed cycloisomerization in trapping the developing carbocation with internal nucleophiles, Shin and co-workers expanded their work to feature iodonium-mediated cyclizations to produce highly functionalized iodocyclohexenes from substituted 1,5-enynes [101]. Substrates of the type shown in Scheme 10 reacted to form exclusively the iodocarbonate products, thus realizing a highly efficient domino reaction that creates two new stereogenic centers and three new bonds.

**Scheme 11:**
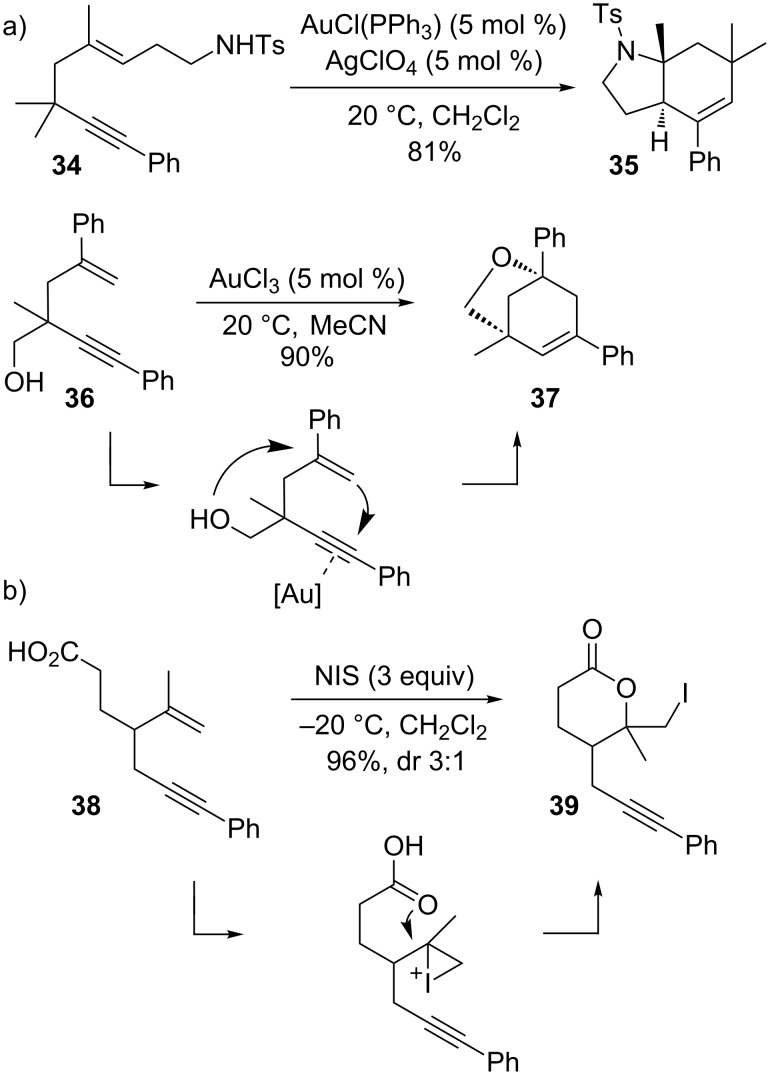
Intramolecular nucleophilic trapping.

Other heteroatoms have also been successfully employed as internal nucleophiles for the trapping of positive charges. In gold-catalyzed cyclizations, Kozmin and co-workers reported the cycloisomerization of 1,5-enynes with nitrogen-tethered, as well as with oxygen-based nucleophiles (e.g., **34→35**; Scheme 11) [102]. It was postulated that the reaction proceeds in a concerted manner, as the double cyclization is highly diastereospecific due to the addition of the nucleophile and alkyne to the alkene being solely *anti.* This reaction is an excellent example that underlines the great potential of the alkynophilicity of the gold metal center. While gold-catalysts make both carbon–carbon and carbon–heteroatom bond formation possible, the analogous process with iodine electrophiles was not realized. Instead, a classical iodolactonization occurred in the reaction of acid **38** with NIS that left the alkyne moiety of the molecule completely untouched [90].

**Scheme 12:**
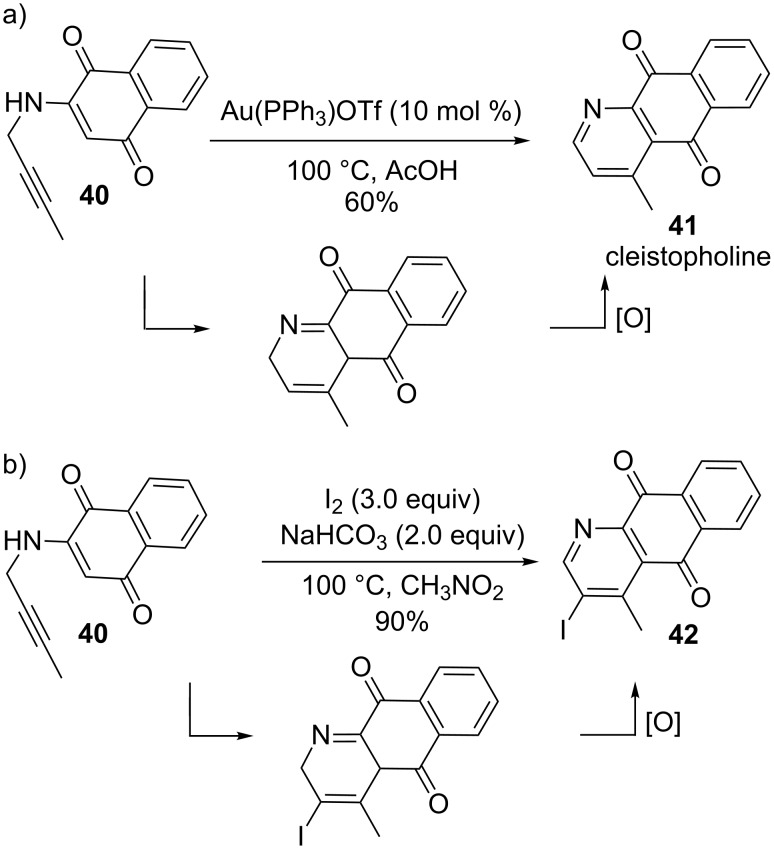
Approach to azaanthraquinones.

**Scheme 13:**
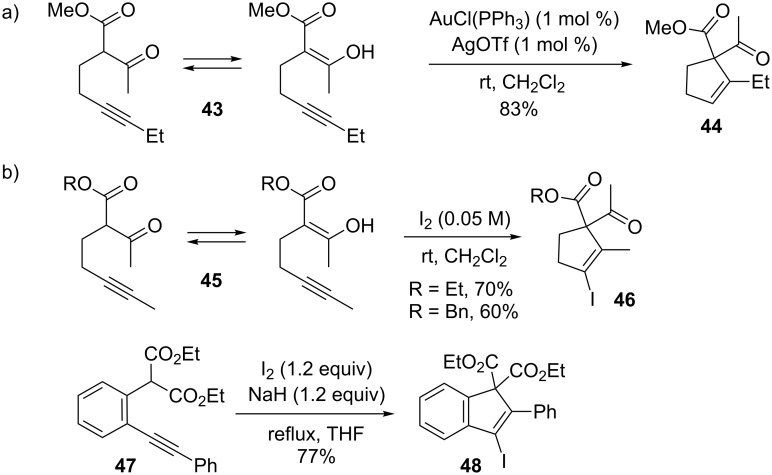
Carbocyclizations with enol derivatives.

Other cyclizations of 1,5-enynes make use of highly nucleophilic enamine or enol moieties, as shown in Scheme 12 and Scheme 13. For example, Wang and co-workers achieved the synthesis of azaanthraquinones from *N*-propargylaminoquinones both by gold- and iodine-triggered processes via 6-endo cycloisomerization modes (Scheme 12) [103–104]. The gold-catalyzed process required a Au(PPh_3_)OTf loading of 10 mol % since an increased reaction time and decreased yield were observed with only 5 mol % of the pre-catalyst. It was found that electron-donating groups on the alkyne terminus facilitated the cyclization while electron-withdrawing substituents hindered it. Under the optimized conditions (10 mol % Au(PPh_3_)OTf, 100 °C), successful synthesis of the alkaloid cleistopholine (**41**) from the aminoquinone **40** was easily achieved after in-situ aromatization of the intermediate to yield the desired compound in 60% yield. Furthermore, Wang and co-workers investigated a totally analogous cycloisomerization sequence using iodine as the electrophile to activate the alkyne, and induce nucleophilic attack by the alkene (Scheme 12b). In this variant, molecular iodine coordinates first with the alkyne **40** analogous to the activation using gold to form an iodonium ion, after which the cyclization proceeds as before to give the final product **42** containing iodine incorporated in the azaanthraquinone. The incorporation of iodine presents great possibilities for further modification and elaboration of the cleistopholine structure.

The nucleophilic properties of enol moieties have also been exploited in the gold-catalyzed intramolecular addition of ß-keto esters to alkynes. For example, Toste and co-workers investigated the previously unreported 5-endo carbocyclization of **43** which involves the cyclization of ß-keto esters onto non-terminal alkynes by use of gold catalysts (Scheme 13) [105], where traditional transition metal-catalyzed Conia-ene type cyclizations are possible only with terminal alkynes [106]. It was proposed that the exclusive formation of the 5-endo product occurred with non-terminal alkynes because 5-exo cyclization, analogous to the traditional Conia-ene mechanism, results in too much strain in the transition state during gold-activation, and therefore 5-endo cyclization is favored in the conversion of acetylenic dicarbonyl compounds. Notably, Toste and co-workers also reported a valuable gold-catalyzed enantioselective variant of the Conia-ene reaction between ß-dicarbonyl compounds and alkynes [107]. Barluenga and co-workers successively developed a 5-endo approach to the iodocarbocyclization between ß-keto esters and non-terminal alkynes (Scheme 13b) [108]. Initially, 1,2-addition of iodine to alkynes such as **45** proved problematic, which was resolved by decreasing the molarity of the iodine in CH_2_Cl_2_ from 0.3 M to 0.05 M. This favored the cyclization process and eliminated competing reaction pathways. It was found that even haloalkynes could be employed in this type of cyclization to give doubly halogenated alkenes. The closely related synthesis of 3-iodo-1*H*-indenes such as **47** via electrophilic cyclization was examined by Wirth and co-workers in 2009 [109]. Following deprotonation by NaH, the iodine activates the alkyne and promotes nucleophilic attack of the stabilized enolate. With NIS as the iodonium source no cyclization was observed after deprotonation and only an α-iodomalonate and starting material were obtained.

#### Diversity-creating transformations

**Scheme 14:**
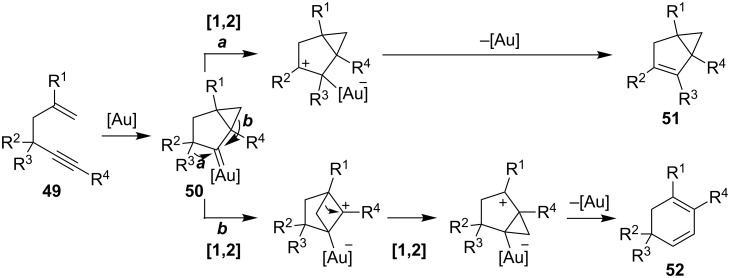
Gold-catalyzed cyclization modes for 1,5-enynes.

There are several gold-catalyzed processes for which there are no corresponding iodine counterparts. In general, most of the processes specific for gold are assumed to proceed via gold carbene intermediates. For example, 1,5-enynes **49** can react in gold-catalyzed domino reactions that include a 1,2-migration as an additional step (Scheme 14). Gold-induced activation of the alkyne followed by cyclization produces a cyclopropyl gold carbene **50** as the key intermediate. Depending on the substitution pattern, two alternative reaction outcomes are possible: The formation of bicyclo[3.1.0]hexane derivatives **51** (path a) or the formation of cyclohexadiene derivatives **52** (path b).

**Scheme 15:**
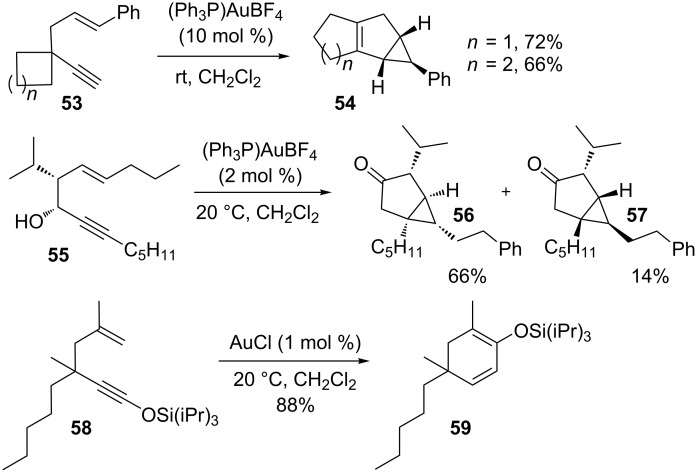
Iodine-induced cyclization of 1,5-enynes.

The catalytic isomerization of 1,5-enynes to bicyclo[3.1.0]hexenes following path a was thoroughly investigated by Toste and co-workers (e.g., **53→54**; Scheme 15) [110]. Transformations of 1,5-enynes that involve 1,2-alkyl migration of R^3^ are strictly limited to compounds that bear a quaternary center (R^3^ = alkyl, R^2^ ≠ H). As shown for the gold(I)-catalyzed reaction of 1,5-enyne **53**, the formation of the bicyclo[3.1.0]hexene **54** is driven by the release of ring strain. Enynes with R^3^ = H undergo exclusively a hydride shift to give the corresponding bicyclo[3.1.0]hexenes of type **51**. By contrast, Gagosz reported the gold-catalyzed cycloisomerization of 4-hydroxylated 1,5-enynes to yield a diverse range of products [111]. The syn-compound **55** reacted with (PPh_3_)AuBF_4_ to give a mixture of diastereomers **56** and **57** in 66 and 14% yield. Under the same reaction conditions, the anti-isomer exhibited reversed selectivity, indicating that the hydroxyl group functions as a possible stereodirecting component in this conversion. However, 1,5-enynes may participate in an alternative reaction pathway via path b (Scheme 14). This is dependent on the substitution pattern and configuration of the enyne and leds to variable product formation. For example, Kozmin and co-workers showed in 2006 that 1,5-enynes can also rearrange to 1,3-cyclohexadienes through a series of 1,2-alkyl shifts (Scheme 15) [112]. In particular, when silyloxy enynes such as **58** were treated with AuCl, the formation of cyclohexadienes was strongly favored, presumably due to the highly stabilized oxonium ion intermediates (path b).

**Scheme 16:**
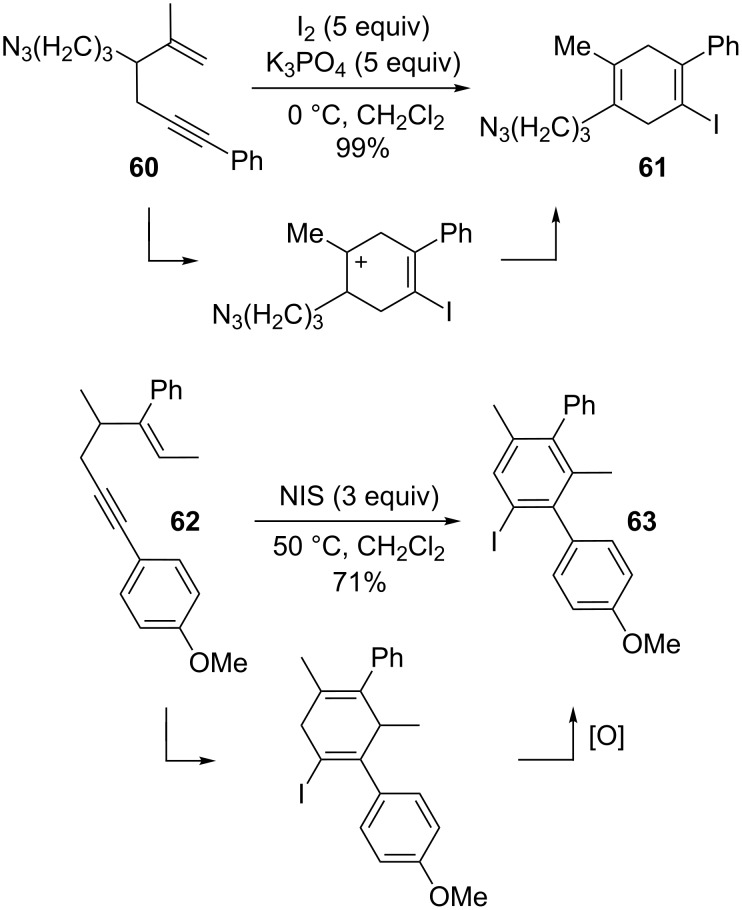
Diverse reactivity of 1,6-enynes.

Essentially, these transformations are not viable with an iodonium source as they do not possess the ability to proceed through simple cationic intermediates. As outlined in Scheme 16 [90,113], classical cationic intermediates and subsequent loss of a proton do lead to iodine-containing 1,4-cyclohexadienes, the formation of which does not include a 1,2-shift. Additionally, the oxidative power of NIS is able to oxidize cyclohexadienes further to aryl systems. It was found that a substituent at C2 was required in both cases to stabilize the carbocation in the cyclic intermediate.

### Carbocyclizations with 1,6-enynes

**Scheme 17:**
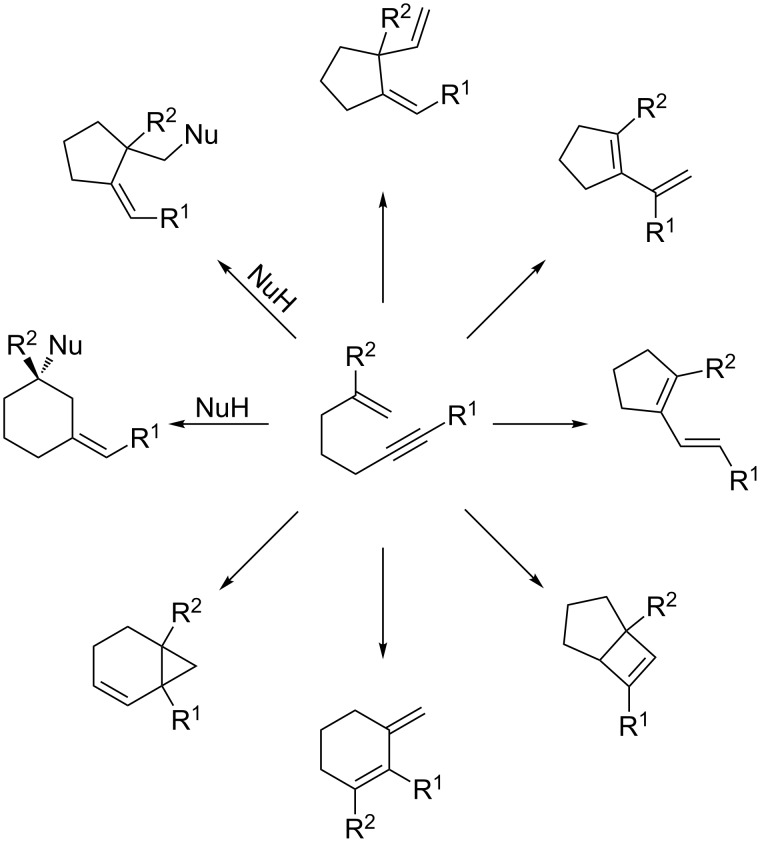
Iodocyclization of 1,6-enynes.

**Scheme 18:**
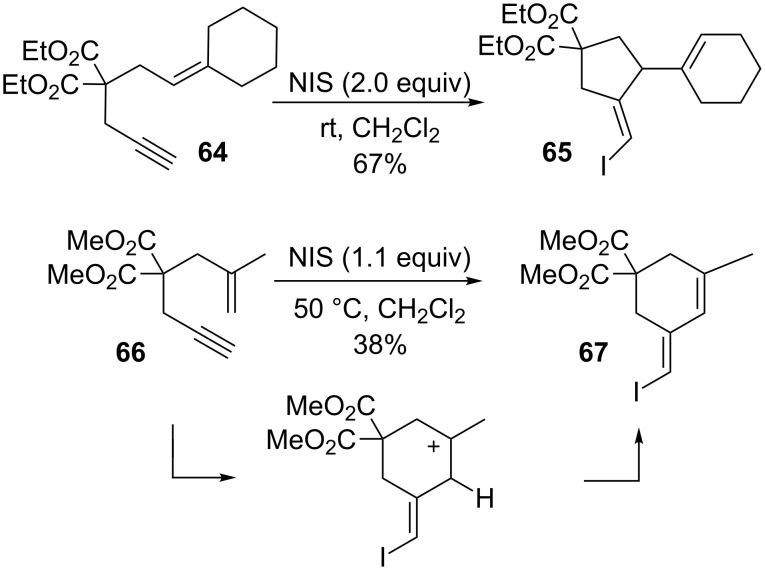
Cyclopropanation of alkenes with 1,6-enynes.

The gold-catalyzed carbocyclizations of 1,6-enynes have been extensively studied. Significant results have been reported by Echavarren and others in recent years and have been extensively reviewed [76–83]. As summarized in Scheme 17, the reactivity of 1,6-enynes displays great variability and a diverse range of product scaffolds are accessible (Scheme 17). From a mechanistic perspective, these cyclizations proceed through gold-carbene intermediates, and the gold in all cases activates the alkyne moiety exclusively. Consequently, most of the gold-catalyzed cyclization modes are not paralleled by iodine-mediated processes. Iodocyclization of 1,6-enynes is known only from very recent examples from Kirsch and co-workers [114]. As shown in Scheme 18, these processes give the cyclization products in only poor yields. With regard to the scope, a terminal alkyne was required for the 6-exo cyclization mode to occur. As with all of the iodine mediated cyclizations discussed above, the cyclization mode is determined by the stability of the intermediate upon carbon–carbon bond formation. It was found that the substitution pattern of the alkene moiety influenced the cyclization mode, whereby a substituent at C2 encourages 6-exo cyclization, and a disubstituted C1 favors 5-exo cyclization.

**Scheme 19:**
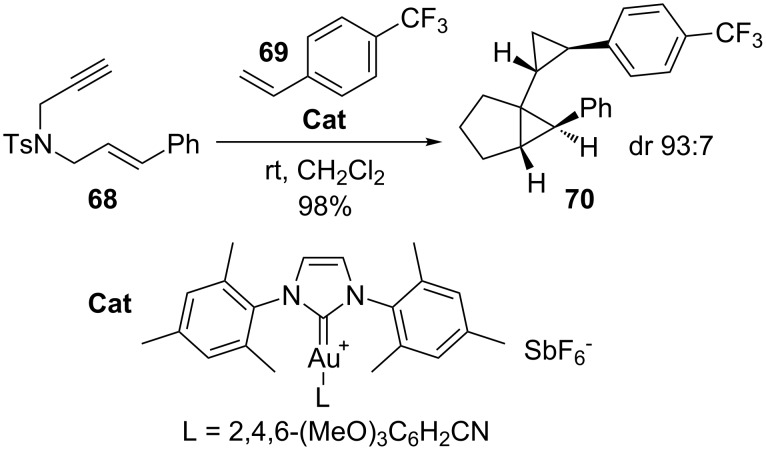
Cyclopropanation of alkenes with 1,6-enynes.

On the other hand, gold-catalyzed domino processes with 1,6-enynes have been shown to proceed in high yields to provide access to carbon skeletons that are not easily synthesized by other approaches. A striking example of the latter is outlined in Scheme 19 [115]. The 1,6-enyne **68** reacts with substituted styrene **69** in the presence of an Au catalyst to afford the cyclopropanation product **70** with excellent diastereoselectivity. This is only one example where gold catalysts open the door to a realm of reactivity that traditional electrophiles can never reach.

## Conclusion

This review was intended to demonstrate that numerous domino processes can be carried out with either gold catalysts or iodine electrophiles to access the same core unit in an analogous manner. While electrophilic cyclization can incorporate iodine into the final product, gold catalysis typically results in hydrogen at the same position due to the protodeauration step required for catalyst regeneration. It is certain that many more reactions that have been described as gold-catalyzed will also be described as iodine mediated in the near future. Apparent in this small survey is also the fact that the electrophilic cyclization mode always proceeds through the most stable cationic intermediates. Therefore, the analogy between gold-catalyzed and iodine mediated reactions only holds true if the gold-catalyzed process makes use of the cationic character of the intermediate. The plethora of gold-catalyzed processes that are based on reactive carbene intermediates will most likely never be matched by electrophilic analogues and will be unique tools for the creation of valuable target compounds.
